# Role of enteroendocrine L‐cells in arginine vasopressin‐mediated inhibition of colonic anion secretion

**DOI:** 10.1113/JP272053

**Published:** 2016-04-28

**Authors:** Ramona Pais, Juraj Rievaj, Claire Meek, Gayan De Costa, Samanthie Jayamaha, R. Todd Alexander, Frank Reimann, Fiona Gribble

**Affiliations:** ^1^ The Wellcome Trust – MRC Institute of Metabolic ScienceMetabolic Research LaboratoriesUniversity of CambridgeAddenbrooke's HospitalCambridgeUK; ^2^ Department of Clinical BiochemistryCambridge University HospitalsAddenbrooke's HospitalCambridgeUK; ^3^ Departments of Paediatrics & PhysiologyFaculty of Medicine & DentistryUniversity of AlbertaEdmontonAlbertaCanada

## Abstract

**Key points:**

- Arginine vasopressin (AVP) stimulates the release of enteroendocrine L‐cell derived hormones glucagon‐like peptide‐1 (GLP‐1) and peptide YY (PYY) *in vitro* from mouse and human colons.
- This is mediated by the AVP receptor 1B, which is highly enriched in colonic L‐cells and linked to the elevation of L‐cell calcium and cAMP concentrations.
- By means of Ussing chambers, we show that AVP reduced colonic anion secretion, although this was blocked by a specific neuropeptide Y receptor Y1 receptor antagonist, suggesting that L‐cell‐released PYY acts locally on the epithelium to modulate fluid balance.
- In human serum samples, PYY concentrations were higher in samples with raised osmolality and copeptin (surrogate marker for AVP).
- These findings describe, for the first time, the role of L‐cells in AVP regulated intestinal fluid secretion, potentially linking together hormonal control of blood volume and blood glucose levels, and thus adding to our understanding of the complex pathways involved in the gut hormonal response to different stimuli.

**Abstract:**

Arginine vasopressin (AVP) regulates fluid balance and blood pressure via AVP receptor (AVPR)2 in the kidney and AVP receptor 1A in vascular smooth muscle. Its role in intestinal function has received less attention. We hypothesized that enteroendocrine L‐cells producing glucagon‐like peptide 1 (GLP‐1) and peptide YY (PYY) may be a target of AVP and contribute to the control of fluid balance. *Avpr1b* expression was assessed by quantitative RT‐PCR on flourescence‐activated cell sorting‐isolated L‐ and control cells and was enriched in colonic L‐cells. AVP stimulated GLP‐1 and PYY release from primary cultured murine and human colonic cells and was associated with elevated calcium and cAMP concentrations in L‐cells as measured in cultures from GLU‐Cre/ROSA26‐GCaMP3 and GLU‐Epac2camps mice. An antagonist of AVPR1B reduced AVP‐triggered hormone secretion from murine and human cells. In Ussing chambers, basolaterally applied AVP reduced colonic anion secretion and this effect was blocked by a specific neuropeptide Y receptor Y1 (NPY1R) antagonist. In human serum, PYY concentrations were higher in samples with raised osmolality or copeptin (a surrogate marker for AVP). In conclusion, we propose that AVP activates L‐cell AVPR1B, causing GLP‐1 and PYY secretion. PYY in turn reduces colonic anion secretion via epithelial NPY1R. Our data suggest L‐cells are active players in the hypothalamic control of intestinal fluid homeostasis, providing a potential link between the regulation of blood volume/pressure/osmolality and blood glucose.

## Key points

- Arginine vasopressin (AVP) stimulates the release of enteroendocrine L‐cell derived hormones glucagon‐like peptide‐1 (GLP‐1) and peptide YY (PYY) *in vitro* from mouse and human colons.
- This is mediated by the AVP receptor 1B, which is highly enriched in colonic L‐cells and linked to the elevation of L‐cell calcium and cAMP concentrations.
- By means of Ussing chambers, we show that AVP reduced colonic anion secretion, although this was blocked by a specific neuropeptide Y receptor Y1 receptor antagonist, suggesting that L‐cell‐released PYY acts locally on the epithelium to modulate fluid balance.
- In human serum samples, PYY concentrations were higher in samples with raised osmolality and copeptin (surrogate marker for AVP).
- These findings describe, for the first time, the role of L‐cells in AVP regulated intestinal fluid secretion, potentially linking together hormonal control of blood volume and blood glucose levels, and thus adding to our understanding of the complex pathways involved in the gut hormonal response to different stimuli.

**Abbreviations**
AVP: arginine vasopressin
AVPR: arginine vasopressin receptor
BIBP: BIBP3226
BMI: body mass index
CFP: cyan fluorescent protein
FACS: flourescence‐activated cell sorting
GLP‐1: glucagon‐like peptide‐1
NPY1R: neuropeptide Y receptor Y1
osmo: osmolality
PYY: peptide YY
RFP: red fluorescent protein
SSR: SSR‐149415
YFP: yellow fluorescent protein

## Introduction

The regulation of fluid balance is vital for human health and is under the control of a number of hormones, including arginine vasopressin (AVP; anti‐diuretic hormone). AVP is produced in the supraoptic and paraventricular nuclei in the hypothalamus and travels along the neuronal axons to the posterior pituitary gland where it is released into the bloodstream. Hypotension and hyperosmolarity stimulate AVP release, such as might occur during dehydration or fluid loss. AVP can bind to three subtypes of G‐protein coupled AVP receptor (AVPR), namely AVPR1A, AVPR1B and AVPR2, which are differently expressed in various tissues, exerting a variety of actions (Koshimizu *et al*. 2012). AVP acts upon the kidney via AVPR2 to increase the water permeability of the collecting ducts, thereby promoting water reabsorption and facilitating the production of concentrated urine. AVP acts upon smooth muscle via AVPR1A to increase peripheral vascular resistance and raise blood pressure during hypovolaemic shock. Vasopressin‐induced platelet aggregation also appears to be mediated via AVPR1A (Launay *et al*. 1987). The role of the AVPR1B is less clear, although it appears to be present in the pituitary and adrenal medulla and promotes the release of adrenocorticotrophic hormone and catecholamines, respectively.

Another potential site of action for AVP might be the gastrointestinal tract, which is the main route of water entry into the body and an important site of fluid loss in pathological states characterized by vomiting or diarrhoea. Intestinal water balance is known to be hormonally regulated. Both glucocorticoids and mineralocorticoids stimulate intestinal sodium and water absorption, with mineralocorticoids predominantly affecting the distal part of the colon, whereas glucocorticoids act along the length of both the small and large bowel (Levitan & Ingelfinger, 1965; Charney *et al*. 1975; Binder *et al*. 1989; Turnamian & Binder, 1989). Human and rodent intestine both express vasopressin receptors (Monstein *et al*. 2008; Ferrier *et al*. 2010). The effect of AVP on intestinal water and salt balance has been investigated previously. *In vitro*, AVP increased colonic sodium, chloride and water absorption at the same time as decreasing anion secretion (Grady *et al*. 1970; Bridges *et al*. 1983; Bridges *et al*. 1984; Knobloch *et al*. 1989; Vicentini‐Paulino, 1992). The role of AVP in altering intestinal water balance was also supported by its ability to increase the pericryptal sodium concentration and upregulate aquaporin‐2 expression in the rat distal colon (Cristià *et al*. 2007). However, the exact mechanism of intestinal AVP action has never been examined. Furthermore, a few *in viv*o studies failed to confirm that AVP stimulates intestinal salt and water absorption as observed *in vitro*, possibly as a result of the effects of AVP on gastrointestinal vasculature and/or motility (Levitan & Mauer, 1968; Dennhardt *et al*. 1979).

Besides being a hormonal target, the intestine also produces multiple hormones such as gastrin, ghrelin, cholecystokinin, glucagon‐like peptide 1 (GLP‐1) and peptide YY (PYY). These hormones are secreted from mucosal enteroendocrine cells and co‐ordinate nutrient absorption and disposal, hunger and satiety (Gribble & Reimann, 2015). GLP‐1 and PYY are secreted by L‐cells, which are found in high numbers in the distal ileum and colon. They appear to be released under similar conditions; for example, when nutrients in the gastrointestinal lumen make contact with L‐cells after a meal (Pais *et al*. 2016 *a*). Although the main role for GLP‐1 appears to be the regulation of glucose homeostasis, the clinical use of GLP‐1 receptor agonists for the treatment of type 2 diabetes has revealed their effects on heart rate and blood pressure, probably reflecting the expression of GLP1R in the heart, brainstem, kidney and arterial vasculature (Pyke *et al*. 2014; Richards *et al*. 2014). PYY, besides its effects on appetite and gastrointestinal motility, is also a strong inhibitor of intestinal water and anion secretion (Cox, 2007 *a*,*b*). In humans, PYY reduces intestinal secretion (Playford *et al*. 1990) via actions on the neuropeptide Y receptors Y1 and Y2 (NPY1R and NPY2R) (Cox, 2008). Thus, L‐cells have the potential to influence both glucose homeostasis and intestinal water balance. The present study aimed to investigate whether hormone secretion from L‐cells was affected by AVP release *in vivo* and *in vitro* and the systemic consequences.

## Methods

### Solutions and compounds

All compounds were purchased from Sigma‐Aldrich (Poole, UK) unless otherwise stated. BIBP3226 trifluoroacetate was purchased from Bioquote (York, UK). SSR‐149415 (SSR) was purchased from Axon Medchem BV (Groningen, The Netherlands). The composition of standard bath solution used in the secretion and imaging experiments was: 4.5 mmol l^−1^ KCl, 138 mmol l^−1^ NaCl, 4.2 mmol l^−1^ NaHCO_3_, 1.2 mmol l^−1^ NaH_2_PO_4_, 2.6 mmol l^−1^ CaCl_2_, 1.2 mmol l^−1^ MgCl_2_ and 10 mmol l^−1^ Hepes (adjusted to pH 7.4 with NaOH). For experiments where CoCl_2_ was used, carbonates and phosphates were omitted from the saline buffer and the osmolality was compensated with additional NaCl (143 mmol l^−1^ total).

The composition of Ringer solution used in the Ussing chamber experiments was: 120 mmol l^−1^ NaCl, 3 mmol l^−1^ KCl, 0.5 mmol l^−1^ MgCl_2_, 1.25 mmol l^−1^ CaCl_2_, 23 mmol l^−1^ NaHCO_3_ and 10 mmol l^−1^ glucose.

### Ethical approval

All animal procedures were approved by the University of Cambridge Animal Welfare and Ethical Review Body and conformed to the Animals (Scientific Procedures) Act 1986 Amendment Regulations (SI 2012/3039). The work was performed under the UK Home Office Project License 70/7824. The ethical principles under which *The Journal of Physiology* operates were fully understood and the work complies with the animal ethics checklist. The experiments presented did not involve regulated procedures, other than the breeding of transgenic mice, or *in vivo* intervention studies. Male and female mice, aged 3–6 months on a C57BL6 background, were housed in individually‐ventilated cages with access to water and chow available *ad libitum*. Mice were killed by cervical dislocation and the intestinal tissue was used in the experiments.

### Transgenic mice

GLU‐Venus mice have been described previously (Reimann *et al*. 2008). GLU‐Cre mice were created using a bacterial artificial construct, as described previously (Parker *et al*. 2012), and express *Cre* recombinase under the control of the proglucagon promoter. Labelling of intestinal L‐cells with a red fluorescent protein (RFP) was achieved by crossing GLU‐Cre with ROSA26tdRFP reporter mice (Luche *et al*. 2007). To monitor calcium fluctuations in L‐cells, these mice were crossed with commercially available ROSA26‐GCaMP3 reporter mice (Zariwala *et al*. 2012) (Jax stock 014538) to generate L‐cell specific expression of this genetically encoded Ca^2+^ sensor. To monitor cAMP changes in L‐cells, transgenic mice expressing Epac2camps under the control of the proglucagon promoter were used; the creation of these mice has been described previously (Psichas *et al*. 2016).

### Primary murine colonic crypt culture

Colonic crypts were isolated and cultured as described previously (Reimann *et al*. 2008). Briefly, mice, aged 3–6 months, were killed by cervical dislocation and the colon was excised. Luminal contents were flushed thoroughly with phosphate‐buffered saline and the outer muscle layer was removed. Tissue was minced and digested with collagenase type XI (0.4 mg ml^−1^) and the cell suspension plated onto Matrigel (BD Bioscience, Oxford, UK) pre‐coated 24‐well plates for GLP‐1 secretion experiments or on 35 mm glass bottomed dishes (Mattek Corporation, Ashland, MA, USA) for calcium and cAMP imaging.

### Preparation of crypt cultures from human colons

The present study was approved by the Research Ethics Committee under license number 09/H0308/24. Fresh surgical specimens of human colon were obtained from Tissue Bank at Addenbrooke's Hospital (Cambridge, UK) stored at 4°C and processed within hours of surgery. The procedure was similar to that for mouse tissue, with the exception that the tissue was digested with a higher concentration of collagenase XI (0.5 mg ml^−1^) (Habib *et al*. 2013).

### GLP‐1 and PYY secretion assays

Some 18–24 h after plating, cells were washed and incubated with test agents made up in standard bath solution supplemented with 0.1% BSA for 2 h at 37°C. At the end of the incubation, supernatants were collected and centrifuged at 400 g for 5 min and snap frozen on dry ice. Cells were lysed with lysis buffer containing (mmol l^–1^): 50 Tris‐HCl, 150 NaCl, 1% IGEPAL‐CA 630, 0.5% deoxycholic acid and complete EDTA‐free protease inhibitor cocktail (Roche, Burgess Hill, UK) to extract intracellular peptides and centrifuged at 10,000 g for 10 min and snap frozen. GLP‐1 and PYY were measured using a total GLP‐1 and total PYY assay (MesoScale Discovery, Gaithersburg, MD, USA) and supernatant concentrations were expressed as a percentage of the total (secreted + lysate) GLP‐1 or PYY content of each well.

### Calcium imaging

Intracellular calcium concentrations were monitored from colonic crypt cultures prepared from GluCre/ROSA26‐GCaMP3/ROSA26‐tdRFP mice. L‐cells were identified by RFP fluorescence and changes in intracellular calcium levels were represented by the change in the intensity of GCaMP3 fluorescence excited at 488 nm using a xenon arc lamp and a monochromator (Cairn Research, Faversham, UK). Solutions were perfused continuously at a rate of ∼1 ml min^−1^. Fluorescence in the presence of the test agent was normalized to the respective mean background fluorescence of each cell, measured before the addition and after the washout of the test compound. Data were smoothened with a sliding average across 20 s. Imaging was performed using an IX71 microscope (Olympus) with a 40× oil immersion objective and an OrcaER camera (Hamamatsu Photonics KK, Hamamatsu, Japan). Images were acquired at 1 Hz and analysed, after background subtraction, using MetaFluor software (Molecular Devices, Sunnyvale, CA, USA).

### cAMP Förster resonance energy transfer measurements

Single‐cell measurements of cAMP levels were made using intestinal cultures from transgenic mice expressing the Förster resonance energy transfer‐based sensor *Epac2‐camps* (Nikolaev *et al*. 2004) under the control of the proglucagon promoter. Briefly, primary colonic L‐cells, continuously perfused with saline solution with or without test reagents at a rate of ∼1 ml min^−1^, were visualized with a 40× oil immersion objective on an inverted microscope (Olympus IX71). Excitation at 435 nm was achieved using a xenon arc lamp coupled to a monochromator (Cairn Research) controlled by MetaFluor software (Molecular Devices). Cyan fluorescent protein (CFP) emission at 470 nm and yellow fluorescent protein (YFP) emission at 535 nm were monitored using an Optosplit II beam splitter (Cairn Research) and an Orca‐ER digital camera (Hamamatsu Photonics KK) and expressed as the CFP/YFP fluorescence ratio. Data were smoothened with a sliding average across 30 s. Peak CFP/YFP ratios were determined at baseline (30 s period prior to test condition) and after test reagent application, and the response to test agent was expressed relative to baseline.

### Quantitative RT‐PCR

Populations of Venus‐positive cells (L‐cells) or Venus‐negative cells (non‐L‐cells) of purity >90% were separated from the tissues of GLU‐Venus mice using a MoFlo cell sorter (Beckman Coulter, High Wycombe, UK) as described previously (Reimann *et al*. 2008). RNA was extracted from FACS‐sorted cells using a microscale RNA isolation kit (Ambion, Austin, TX, USA) and was then reverse transcribed to cDNA in accordance with standard protocols. The appropriate amount of first‐strand cDNA template was mixed with specific TaqMan primers (Applied Biosystems, Foster City, CA, USA), water and PCR Master Mix (Applied Biosystems), and a quantitative RT‐PCR was conducted using a 7900HT Fast Real‐Time PCR system (Applied Biosystems). β‐actin was used as the normalization control. The primer/probe pairs used in the present study were: *Avpr1a*: Mm00444092_m1; *Avpr1b*: Mm01700416_m1; and *Avpr2*: Mm01193534_g1 (all from Applied Biosystems). All experiments were performed on at least three cDNAs each isolated from one mouse (*n* = 3 mice).

### Short circuit current (*I*_Sc_) measurement

The most distal part of the colon (∼1.25 cm) was cut open longitudinally, rinsed in Ringer solution and mounted in an Ussing chamber (EM‐LVSYS‐4 system with P2400 chambers and P2404 sliders; Physiologic Instruments, San Diego, CA, USA). Only one preparation was used from each animal. The active epithelial surface was 0.25 cm^2^. Both parts of the Ussing chambers were filled with 3 ml of Ringer solution, maintained at 37°C and continuously bubbled with 5% v/v CO_2_/ 95% v/v O_2_. The transepithelial potential difference was clamped to 0 mV using a DVC 1000 amplifier (WPI, Sarasota, FL, USA) and the resulting short circuit current was recorded via Ag‐AgCl electrodes and 3 mol l^−1^ KCl agarose bridges. The recordings were collected and stored using a Digidata 1440A acquisition system and AxoScope, version 10.4 (Molecular Devices). The transepithelial resistance and short circuit current (*I*_sc_) were allowed to stabilize for at least 30 min before the application of drugs. During this period, transepithelial resistance was assessed by measuring current changes in response to 2 mV pulses lasting 2.5 s, applied every 100 s. After stabilization of the electrical parameters, the drugs applied were: 5 μmol l^−1^ amiloride, 1 μmol l^−1^ BIBP3226 (BIBP) and 1 μmol l^−1^ AVP. A mix of forskolin (10 μmol l^−1^) + IBMX (100 μmol l^−1^) was applied bilaterally at the end of each experiment to confirm the responsiveness/viability of the tissue.

### Human study design

Ethical approval was obtained to measure gut hormone concentrations in human serum samples that were left over after diagnostic testing was completed (REC 14/EE/1247; 1/12/2014). We selected blood samples from patients in which osmolality assessment had been requested for clinical reasons. Serum was collected using a separator tube, centrifuged within 4 h and stored at 4°C prior to analysis. Osmolality was measured using the freeze‐point depression method (Model 3320 Osmometer; Advanced Instruments Inc., Norwood, Massachusetts, USA). Total PYY was measured using a commercially available sandwich immunoassay kit (MesoScale Discovery), which measures both the 1–36 and 3–36 forms of PYY. The analytical range is 28–3000 pg ml^−1^. Inter‐assay coefficients of variation of 7.8‐16.4% were obtained within the physiological range. GLP‐1 was not measured because it is not stable during prolonged storage at 4°C. To estimate the concentration of AVP, we measured copeptin, which is a cleavage product of pro‐AVP released from the pituitary gland in equimolar quantities to AVP but has much greater stability in plasma and is easier to measure (Morgenthaler *et al*. 2006; Morgenthaler, 2010). Copeptin was measured using a sandwich immunoassay (USCN, Houston, TX, USA), which has an analytical range of 15.6‐1000 pg ml^−1^.

We divided the samples into low/high osmolality (osmo) (</>295 mosmol kg^−1^: the upper limit of normal value) and low/high copeptin (</>300 pg ml^−1^; *sensu* Tenderenda‐Banasiuk *et al*. 2014; Masajtis‐Zagajewska *et al*. 2015). The clinical characteristics of the low osmo, high osmo, low copeptin and high copeptin groups, respectively, were: age (median) 77, 50, 69 and 59 years; body mass index (BMI) (median) 21.71, 28.25, 24.02 and 27.11 kg m^–2^; and sex (male/female) 18/14, 20/8, 21/8 and 14/12. Age was significantly higher and BMI significantly lower in the low osmo group compared to the high osmo group. No significant differences in age, BMI or sex were observed between the low and high copeptin groups.

### Statistical analysis

The results are expressed as the mean ± SD unless otherwise indicated. Statistical analysis was performed using Prism, version 5.01 (GraphPad, San Diego, CA, USA). GLP‐1 and PYY secretion data were analysed by two‐way ANOVA, using experimental run and treatment type as variables. Secretion data were heteroscedastic and therefore log transformed. *Post hoc* testing was performed with *post hoc* Dunnett's or Bonferroni tests as indicated. Statistical significance for Ca^2+^ imaging data was assessed by Student's *t* test. Human serum samples that had PYY concentrations below the limit of detection were assigned a value of 28 pg ml^−1^ and the serum PYY levels were analysed non‐parametrically by the Mann–Whitney test. *P* < 0.05 was considered statistically significant.

## Results

### *Avpr1b* expression is enriched in colonic L‐cell population

**Figure 1:**
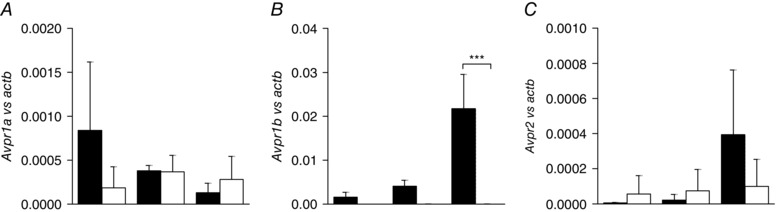
**AVP receptors gene expression in L‐ and non‐L‐cell populations** *Avpr1a* (*A*), *Avpr1b* (*B*) and *Avpr2* (*C*) expression was analysed by quantitative RT‐PCR from FACS‐isolated L‐ (black) and non‐L‐ (white) cells from the upper and lower small intestine (SI) and large intestine (LI) of mice and compared with that of *Actb* in the same sample. A Ct value of 40 was assigned to samples where the target gene was undetected. Data are presented as the geometric mean + upper SD of the 2^ΔCt^ data (*n* ≥ 3 each). Comparisons between L‐ and non‐L‐cells were assessed on non‐transformed ΔCt data using one‐way ANOVA and *post hoc* Bonferroni analysis. ^***^ *P* < 0.001.

The expression of AVP receptors, *Avpr1a* (Fig. 1 *A*), *Avpr1b* (Fig. 1 *B*) and *Avpr2* (Fig. 1 *C*) was assessed by quantitative RT‐ PCR in L‐ and non‐L‐cell populations obtained by FACS sorting of epithelial cell suspensions from the upper and lower half of the mouse small intestine and large intestine. Of the three AVP receptors examined, *Avpr1b* was found to be highly enriched in both small intestinal and colonic L‐cells compared to control cells and was much higher in colonic L‐cell *vs*. small intestinal L‐cell populations (Fig. 1 *B*).

### AVP stimulates GLP‐1 and PYY secretion from mouse colonic cultures

**Figure 2:**
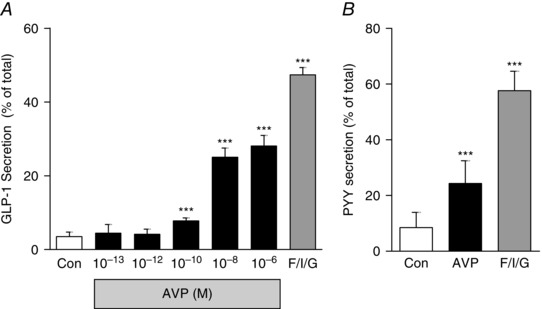
**AVP induced GLP‐1 and PYY secretion from murine colonic cultures** *A*, mixed intestinal cultures generated from mouse colons were incubated for 2 h in saline solution alone (Control; Con) or containing increasing concentrations of AVP or F/I/G, forskolin (10 μmol l^−1^) plus IBMX (10 μmol l^−1^) plus glucose (10 mmol l^−1^). *B*, PYY secretion was measured from mixed cultures incubated with a single concentration of AVP (10 nmol l^−1^). GLP‐1 and PYY secretion is expressed as a percentage of total content. Results are shown as the mean + SD of (*A*) *n* = 9 and (*B*) *n* = 13 or 14 wells with 3 or 4 wells originating from a single mouse. ^***^ *P* < 0.001 compared to controls using one‐way ANOVA followed by *post hoc* Bonferroni analysis on log_10_ transformed data.

To monitor GLP‐1 and PYY secretion, primary crypt cultures were prepared from murine colonic tissue. Cells were stimulated for 2 h with different concentrations of AVP (10^−13^ to 10^−6 ^mol l^−1^) or with a positive control that was a combination of forskolin (10 μmol l^−1^), IBMX (10 μmol l^−1^) and glucose (10 mmol l^−1^). AVP at or above 0.1 nmol l^−1^ caused a significant stimulation of GLP‐1 secretion, which increased from 3.5% to comprise 28% of the total GLP‐1 content with the highest concentration used (Fig. 2 *A*). PYY secretion from murine colonic cultures was also assessed in response to 2 h of incubation with AVP (10 nmol l^−1^) and increased from 9% to comprise 35% of the total PYY content (Fig. 2 *B*).

### AVP elevates intracellular calcium concentrations in colonic L‐cells

**Figure 3:**
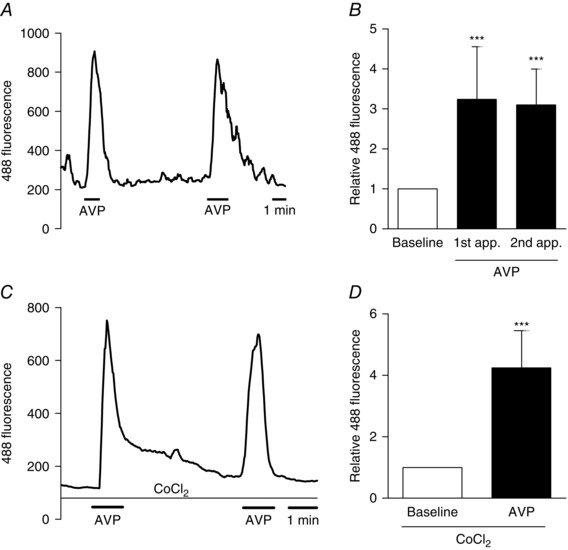
**Intracellular Ca^2+^ changes in colonic L‐cells in response to AVP** Mixed colonic cultures were generated from GLU‐Cre/ROSA26‐GCaMP3/ROSA26‐tdRFP mice and L‐cells were identified by their RFP fluorescence. *A*, representative trace showing calcium response to AVP (10 nmol l^−1^) in an L‐cell as a measure of GCaMP3 fluorescence. *B*, mean GCaMP3 fluorescence changes in L‐cells in response to two applications of AVP in the same cell. *n* = 9 cells and results are the mean + SD. ^***^ *P* < 0.001 compared to baseline by one‐sample Student's *t* test. *C*, representative trace showing calcium response to AVP (10 nmol l^−1^) in the presence of cobalt chloride (CoCl_2_; 5 mmol l^−1^) to block voltage‐gated calcium channels and (*D*) mean calcium changes in L‐cells in response to AVP (10 nmol l^−1^) in the presence of CoCl_2_ (*n* = 6 cells). Results are shown as the mean + SD. ^***^ *P* < 0.001 compared to baseline by a one‐sample Student's *t* test.

To investigate the molecular mechanisms activated by exposure of L‐cells to AVP, calcium fluctuations were monitored in primary L‐cells cultured from the colon of GLU‐Cre/ROSA26‐GCaMP3 mice. These mice express the GCaMP3 protein, a genetically encoded Ca^2+^ sensor, specifically in L‐cells and hence allow intracellular calcium levels to be monitored by exciting the GFP moiety of GCaMP3. AVP at a concentration of 10 nmol l^−1^ caused reversible and reproducible elevations of GCaMP3 fluorescence, which is indicative of an increase in intracellular calcium in colonic L‐cells (Fig. 3 *A* and *B*).

Intracellular calcium increase can either be caused by release of calcium from intracellular stores or influx of extracellular calcium via the opening of voltage‐gated calcium channels in the plasma membrane. To distinguish these possibilities, calcium imaging experiments were performed in the presence of cobalt chloride (CoCl_2_), a general voltage‐gated calcium channel blocker that impairs L‐cell calcium responses to depolarizing stimuli such as KCl (Pais *et al*. 2016 *b*). Cytoplasmic calcium responses to AVP were still observed in the presence of CoCl_2_ (5 mmol l^−1^) (Fig. 3 *C* and *D*), suggesting a lack of dependence on voltage‐gated calcium channels, which is consistent with the known Gq coupled nature of the AVPR1B.

### AVP elevates intracellular cAMP concentrations in colonic L‐cells

**Figure 4:**
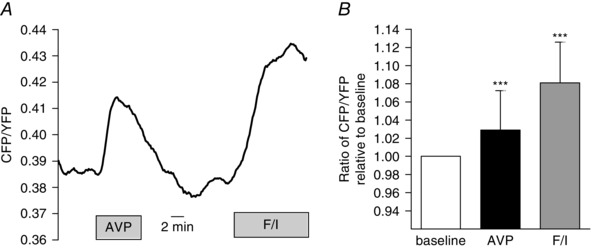
**Intracellular cAMP changes in colonic L‐cells in response to AVP** Mixed colonic cultures were generated from mice expressing Epac2camps in L‐cells. *A*, representative trace of cAMP response in an L‐cell as measured by the CFP/YFP ratio in the presence of AVP (10 nmol l^−1^) and F/I, forskolin (10 μmol l^−1^) + IBMX (100 μmol l^−1^). *B*, mean CFP/YFP changes in L‐cells after the addition of AVP (*n* = 24) and F/I (*n* = 23). Results are shown as the mean + SD. ^***^ *P* < 0.001 compared to baseline by one‐sample Student's *t* test.

AVPR1B has been reported to activate both Gq‐inositol phosphate and Gs‐cAMP pathways, depending on the ligand and the localization within specialized compartments on the plasma membrane (Orcel *et al*. 2009). We tested the effect of AVP on intracellular cAMP levels in single L‐cells in primary cultures prepared from transgenic mice expressing the Förster resonance energy transfer‐based sensor *Epac2‐camps* under the control of the proglucagon promoter. In colonic cultures, AVP (10 nmol l^−1^) treatment resulted in an acute increase in the CFP/YFP fluorescence ratio, which is indicative of an elevation of cytosolic cAMP in L‐cells (Fig. 4).

### Antagonist of AVPR1B reduces AVP‐stimulated GLP‐1 and PYY secretion

**Figure 5:**
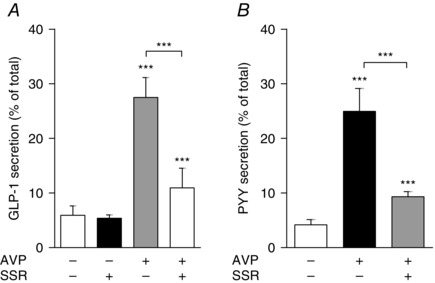
**Antagonist of AVPR1B reduces GLP‐1 and PYY secretion from murine colonic cultures** GLP‐1 (*A*) and PYY (*B*) secretion was measured from cultures treated with AVP (10 nmol l^−1^) in the presence or absence of SSR (1 μmol l^−1^), a selective AVPR1B antagonist. Where applicable, wells were pre‐treated with SSR, 30 min before the administration of AVP. GLP‐1 and PYY secretion is expressed as a percentage of total content. Results are shown as the mean + SD; *n* = 9–17 wells with 3 or 4 wells originating from a single mouse. ^***^ *P* < 0.001 compared to controls or as indicated using one‐way ANOVA followed by *post hoc* Dunnett's test or Bonferroni analysis on log_10_ transformed data.

To test the functional importance of AVPR1B, we performed GLP‐1 and PYY secretion experiments in the presence of SSR (i.e. a selective vasopressin receptor antagonist with specificity for the AVPR1B subtype) (Serradeil‐Le Gal *et al*. 2002; Serradeil‐Le Gal *et al*. 2005). In mouse colonic cultures, SSR (1 μmol l^−1^) significantly reduced AVP triggered GLP‐1 and PYY release by ∼70% and ∼80%, respectively (Fig. 5). SSR on its own, however, had no effect on GLP‐1 secretion (Fig. 5 *A*).

### AVP stimulates GLP‐1 and PYY secretion from human colonic cultures

**Figure 6:**
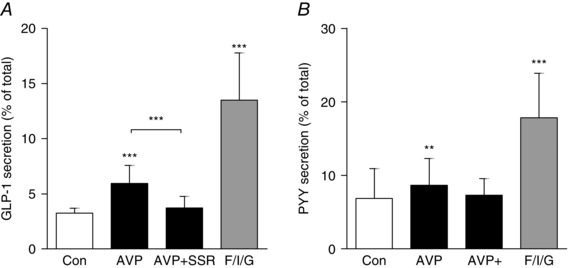
**GLP‐1 and PYY secretion from human colonic cultures** Mixed intestinal cultures were generated from fresh human colonic tissues and stimulated with AVP (10 nmol l^−1^) with or without the AVPR1B antagonist SSR (1 μmol l^−1^). As in Fig. 5, wells that were co‐incubated with AVP + SSR received a 30 min pre‐treatment with the inhibitor. GLP‐1 (*A*) and PYY (*B*) secretion is expressed as a percentage of total content. Results are shown as the mean + SD of (*A*) *n* = 10–20 wells and (*B*) *n* = 8–18 wells with 3 or 4 wells originating from a single tissue sample. ^**^ *P* < 0.01 and ^***^ *P* < 0.001 compared to controls or as indicated using one‐way ANOVA followed by *post hoc* Dunnett's test or Bonferroni analysis on log_10_ transformed data.

To determine whether AVP has an effect on gut hormone secretion in humans, we used crypt cultures prepared from fresh human colon specimens obtained within a few hours of surgery. GLP‐1 and PYY concentrations were measured in supernatants and cell lysates after 2 h of incubation in the presence of 10 nmol l^−1^ AVP. AVP significantly stimulated GLP‐1 and PYY secretion by ∼2‐fold and 1.5‐fold, respectively (Fig. 6). As in the murine cultures, SSR (1 μmol l^−1^) significantly blocked the effect of AVP on GLP‐1 release, suggesting that stimulation is mediated via AVPR1B in humans (Fig. 6 *A*). The reduction of PYY release by SSR did not reach statistical significance in human cultures (Fig. 6 *B*).

### Anti‐secretory effect of AVP in the mouse colon

**Figure 7:**
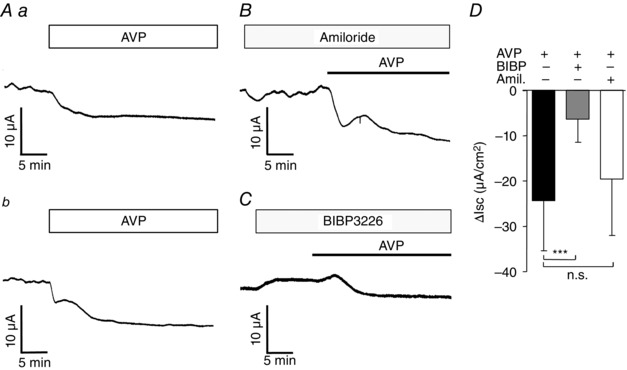
**AVP‐induced effect on short circuit current in the mouse distal colon** *A*, traces showing changes in short circuit current recordings (*I*_sc_) from mouse distal colon mounted in Ussing chambers after basolateral application of AVP (1 μmol l^−1^). Both simple (*a*) and more complicated kinetics (*b*) are depicted. *B*, *I*_sc_ changes from colon tissue as in (*A*) but with the additional presence of apical amiloride (5 μmol l^−1^) to block ENaC‐dependent sodium absorption. *C*, *I*_sc_ changes from colon tissue as in (*A*) but with the additional presence of basolateral NPY1R antagonist BIBP (1 μmol l^−1^). *D*, mean changes in Isc after application of AVP alone or in the presence of BIBP or amiloride (Amil.). Δ*I*_sc_ was calculated as the difference between the means of short circuit currents from the 2–5 min period before and 15 min period after the application of AVP. Data are the mean ± SD from five to nine tissue preparations, normalized for a surface area of 1 cm^2^. ^***^ *P* < 0.001 compared to AVP application alone using one‐way ANOVA followed by *post hoc* Bonferroni analysis on non‐transformed data.

We employed Ussing chambers to study the functional relevance of AVP in the mouse colon. Basolateral addition of AVP (1 μmol l^−1^) caused a reduction in the short circuit current (*I*_sc_) without any obvious change in the transepithelial resistance. In five of nine tissue preparations, the *I*_sc_ change followed a simple curve, reaching a plateau several minutes after the addition of AVP (Fig. 7 *Aa*). The other samples showed more complicated kinetics, with an initial drop in *I*_sc_ and then a period of stabilization or even a slight increase in *I*_sc_, followed by a second fall (Fig. 7 *Ab*). In all cases, *I*_sc_ remained depressed for at least 20 min after AVP addition. Pre‐treatment with apically added amiloride (5 μmol l^−1^) caused a decrease of *I*_sc_ that sometimes partially recovered, reaching a new plateau in 1–5 min. The mean *I*_sc_ decrease as a result of amiloride was 4.37 μA cm^–2^. Subsequent application of AVP, 10–12 min after amiloride, caused a further decrease in *I*_sc_, which was no different from the decrease caused by AVP without any pretreatment (Fig. 7 *B* and *D*). These findings suggest that the effect of AVP was a result of the decrease in electrogenic anion secretion and was not attributable to an interaction with ENaC‐dependent sodium absorption. The results are in agreement with previously published results from Ussing chamber recordings (Bridges *et al*. 1984; Xue *et al*. 2014). Pre‐treatment with the basolaterally added specific NPY1R antagonist BIBP caused an increase of *I*_sc_, reaching a new plateau in 3–6 min. The mean *I*_sc_ increase as a result of BIBP was 7.84 μA cm^–2^. Subsequent application of AVP, 10–12 min after BIBP, triggered a significantly smaller drop in *I*_sc_ compared to when AVP was applied without BIBP pretreatment, confirming a role of the PYY receptor NPY1R in AVP‐mediated changes of colonic transepithelial ion movement (Fig. 7 *C* and *D*).

### Human plasma PYY levels

**Figure 8:**
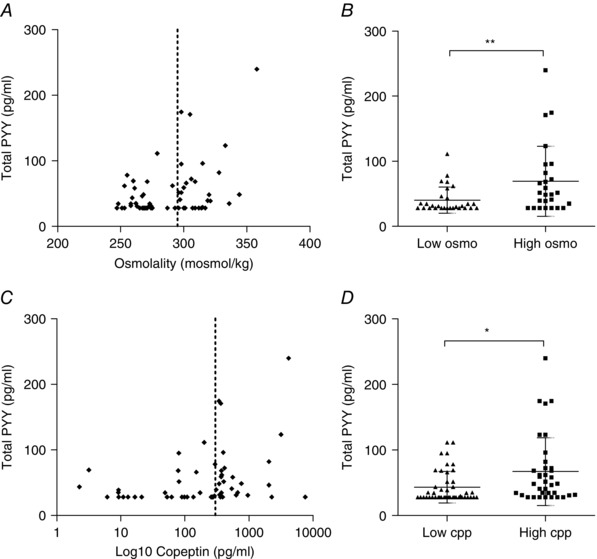
**Plasma PYY measurements in human serum samples** *A*, scatter plot showing total PYY (pg ml^–1^) plotted against osmolality (mosmol kg^–1^) in human serum samples. Samples were divided into low osmo and high osmo groups with a cut‐off limit set at 295 mosmol kg^–1^ (vertical dashed line). *B*, total PYY (pg ml^–1^) was plotted for the two groups: low osmo (circles) and high osmo (squares). *C*, scatter plot showing total PYY (pg ml^–1^) plotted against copeptin (pg ml^–1^) in human serum samples. Copeptin (cpp) was measured as a surrogate marker for AVP in the serum. Samples were divided into a low and high group with a cut‐off set at 300 pg ml^−1^ (vertical dashed line) and (*D*) total PYY (pg ml^–1^) was plotted for the two groups: low cpp (circles) and high cpp (squares). Statistical comparison was made for PYY in the high and low groups by the Mann–Whitney test. ^*^ *P* < 0.05 and ^**^ *P* < 0.01 for low and high groups.

To assess whether PYY release is influenced by AVP in humans, we measured PYY and copeptin concentrations in anonymized human serum samples that had been sent for clinical measurement of osmolality. Copeptin, similar to AVP, is produced by post‐translational processing of pre‐provasopressin, although it is more stable in stored serum samples and can be used as an indirect measure of AVP concentrations (Morgenthaler *et al*. 2006). We hypothesized that serum PYY concentrations would be elevated in conditions of elevated copeptin or osmolality. Figure 8 *A* and *B* shows the scatter of PYY concentrations plotted against osmolality and copeptin. Because we do not predict a linear effect on PYY, we divided the samples into low/high osmolality (</>295 mosmol kg^−1^: the upper limit of normal value) and low/high copeptin (</>300 pg ml^−1^; *sensu* Tenderenda‐Banasiuk *et al*. 2014; Masajtis‐Zagajewska *et al*. 2015). PYY was significantly higher in both the high osmolality and high copeptin groups (Fig. 8 *C* and *D*).

## Discussion

In the present study, we have shown, for the first time, that AVP stimulates GLP‐1 and PYY secretion from colonic L‐cells isolated from mice and humans. It acts via the Gq/Gs coupled receptor AVPR1B, which was found to be highly expressed in colon and was particularly enriched in L‐cells over the neighbouring epithelial cells. In support of this idea, a selective antagonist of AVPR1B significantly decreased AVP‐stimulated hormone release in both mouse and human colonic cultures. Because the action of AVP was not completely abolished by the antagonist, however, we cannot exclude the possibility that the alternative receptors, AVPR1A and AVPR2, also play a role. *In vitro*, AVP‐stimulation of L‐cells raised both intracellular cAMP and calcium and triggered the secretion of both PYY and GLP‐1. One of the known actions of PYY is mediated via Y1 receptors located on neighbouring enterocytes, reducing anion and water secretion to the colonic lumen and ultimately altering local intestinal water balance in the direction of water conservation. This proposed mechanism provides an explanation for some of the previously observed local *in vitro* effects of AVP in the colon, notably a decrease in short circuit current, a decrease in chloride secretion, and an increase in net water and anion absorption (Bridges *et al*. 1983; Bridges *et al*. 1984; Vicentini‐Paulino, 1992; Xue *et al*. 2014). Given the amount of water secreted into the gastrointestinal tract, which is estimated to be ∼8 litres/24 h in humans (Barrett & Keely, 2000), it is possible that the modulation of intestinal secretion through this pathway could have an impact on the systemic circulation. Indeed, i.v. infusion of high doses of PYY to healthy volunteers increased both diastolic and systolic blood pressure (Adrian *et al*. 1986). Our data additionally suggest that serum osmolality and AVP levels influence circulating PYY concentrations in humans, raising the possibility of more systemic consequences of AVP‐triggered secretion from intestinal L‐cells. Age and BMI were significantly different in the low *vs*. high osmolality but not between the copeptin groups, with the low osmo group having both a lower BMI and lower PYY than the high osmo group. Because previous studies reported that PYY levels are inversely related to BMI (Batterham *et al*. 2003), the BMI difference probably does not account for the difference in PYY observed in the present study. Also, there was no influence of age on PYY in healthy population (MacIntosh *et al*. 1999). More experiments will be needed to determine whether the described modulation of L‐cells by AVP is involved in the control of physiological hormonal blood volume/pressure and glucose homeostasis or is solely aimed at the local regulation of intestinal water and ionic movements.

Although it is relatively straightforward to appreciate a possible role of AVP‐induced PYY release, we can only speculate about the purpose of the simultaneous GLP‐1 secretion. A single study has reported anionic secretory responses of murine intestinal epithelium to endogenously released GLP‐1, as well as to application of the GLP1R agonist exendin‐4 (Joshi *et al*. 2013). Other studies, however, did not report any response to GLP‐1 in guinea‐pig ileum, even at high concentrations (Baldassano *et al*. 2012). Similarly, we did not observe any changes in basal short circuit current in response to either GLP‐1 or exendin‐4 stimulation in preparations from the murine distal colon mounted in Ussing chambers (data not shown). This obviously does not preclude the possibility that GLP‐1 might affect other parts of the intestine or do so under different experimental conditions. In another study, GLP‐1 was shown to suppress epithelial secretory responses to electrical stimulation of submucosal neurons (Baldassano *et al*. 2012). This raises the interesting hypothesis that co‐released PYY and GLP‐1 possess complementary properties with respect to intestinal salt and water balance, inhibiting basal (PYY) as well as neuronally stimulated (GLP‐1) anion secretion. Indeed, AVP itself was also shown to inhibit neuronally stimulated secretory responses (Knobloch *et al*. 1989). However, we did not observe any exendin‐4 related inhibition of secretory responses to electrical field stimulation in our preparations from the mouse distal colon (data not shown). There is also an intriguing possibility that AVP‐induced GLP‐1 secretion increases intestinal water uptake via its stimulatory effect on the hypothalamic‐pituitary‐adrenal axis. Indeed, peripheral administration of GLP‐1 or exendin‐4 increased levels of both corticosterone and aldosterone in rats, whereas bolus i.v. GLP‐1 increased systemic levels of cortisol in humans (Gil‐Lozano *et al*. 2010; Gil‐Lozano *et al*. 2014). Prolonged administration of exendin‐4 or other GLP‐1 receptor agonists was also associated with increased corticosterone levels and increased water intake in rodents (Gil‐Lozano *et al*. 2013; Krass *et al*. 2015). Glucocorticoids are known to increase intestinal sodium and water absorption from small and large intestine as a result of their effect on Na^+^/K^+^ ATPase, NHE3 and SGLT1 activity. Under this mechanism, the effects of AVP‐stimulated GLP‐1 and PYY secretion would also be complementary, with PYY reducing anion and water secretion via its direct short term effect on enterocytes, whereas GLP‐1 increases sodium and water absorption via its longer‐term modulation of glucocorticoid levels. Of note, ablation of AVPR1B activity by antagonists or by use of knockout animals leads to decreased levels of glucocorticoids in the circulation, although, here, a central direct action of AVP independent of GLP‐1 has been postulated (Koshimizu *et al*. 2012).

Other than its role in water retention and blood pressure control, AVP is increasingly recognized as an active player in the regulation of glucose homeostasis. AVPR1B‐mediated GLP‐1 release from intestinal L‐cells could provide an additional level of complexity to the direct stimulatory effect of AVP on pancreatic insulin and glucagon secretion (Lee *et al*. 1995; Yibchok‐Anun *et al*. 2000; Abu‐Basha *et al*. 2002). For example, the elevated AVP levels observed in uncontrolled diabetes (Vokes *et al*. 1987) might not only maintain fluid balance, but also stimulate insulin secretion by promoting GLP‐1 release, thereby counteracting the hyperglycaemic state.

In summary, the data obtained in the present study show, for the first time, the role of L‐cells in AVP regulated intestinal fluid secretion, potentially linking the hormonal control of blood volume and blood glucose levels. Although therapies based on GLP‐1 physiology have been highly successful in the treatment of type 2 diabetes, the alteration of intestinal salt and water balance has recently been recognized as a promising target for novel drugs against hypertension, heart failure, end‐stage renal disease and chronic kidney disease (Spencer *et al*. 2014). Thus, clarifying the exact role of L‐cells in the cross‐talk between blood volume/pressure and blood glucose control could be of potential interest with respect to our understanding of the range of diseases currently posing a significant burden on modern society.

## Additional information

### Competing interests

The authors declare that they have no competing interests.

### Author contributions

All experiments were conducted in the laboratories of FMG and FR at the Wellcome Trust‐MRC Institute of Metabolic Science, Metabolic Research Laboratories, University of Cambridge, Addenbrooke's Hospital, Cambridge, UK. RP conceived and designed the work; acquired, analysed and interpreted the data; and drafted and wrote the manuscript. CM and JR designed the work; acquired, analysed and interpreted the data; and drafted the manuscript. GDC and SJ acquired and analysed data and revised the manuscript for intellectual content. RTA interpreted the data for the work and revised the manuscript critically for intellectual content. FMG and FR conceived and designed the work; analysed and interpreted the data; and drafted and revised the manuscript critically for important intellectual content. We confirm that the final version of the manuscript has been read and approved by all named authors and we take responsibility for all aspects of the work to ensure that questions related to the accuracy or integrity of any part of the work are appropriately investigated and resolved. Furthermore, all persons designated as authors qualify for authorship, and all those who qualify for authorship are listed.

### Funding

This work was funded by grants from the Wellcome Trust (106262/Z/14/Z, 106263/Z/14/Z), the MRC Metabolic Diseases Unit (MRC_MC_UU_12012/3) and Full4Health (FP7/2011‐2015, grant agreement no 266408). Juraj Rievaj was initially supported by an EFSD Albert Renold Travel Fellowship.
